# Research on Influencing Factors and Classification of Patients With Mild and Severe COVID-19 Symptoms

**DOI:** 10.3389/fcimb.2021.670823

**Published:** 2021-08-18

**Authors:** Xiaoping Chen, Lihui Zheng, Shupei Ye, Mengxin Xu, YanLing Li, KeXin Lv, Haipeng Zhu, Yusheng Jie, Yao-Qing Chen

**Affiliations:** ^1^College of Mathematics and Statistics & FJKLMAA, Fujian Normal University, Fuzhou, China; ^2^Pulmonary and Critical Care Medicine, The Third People’s Hospital of Dongguan City, Dongguan, China; ^3^School of Public Health (Shenzhen), Sun Yat-sen University, Shenzhen, China; ^4^Department of Infectious Diseases, The Ninth People’s Hospital of Dongguan City, Dongguan, China; ^5^Department of Infectious Diseases, The Third Affiliated Hospital of Sun Yat-sen University, Guangzhou, China

**Keywords:** COVID-19, mild and severe, clinical characteristic, Lasso-Logistic regression, random forest

## Abstract

**Objective:**

To analyze the epidemiological history, clinical symptoms, laboratory testing parameters of patients with mild and severe COVID-19 infection, and provide a reference for timely judgment of changes in the patients’ conditions and the formulation of epidemic prevention and control strategies.

**Methods:**

A retrospective study was conducted in this research, a total of 90 patients with COVID-19 infection who received treatment from January 21 to March 31, 2020 in the Ninth People’s Hospital of Dongguan City were selected as study subject. We analyzed the clinical characteristics of laboratory-confirmed patients with COVID-19, used the oversampling method (SMOTE) to solve the imbalance of categories, and established Lasso-logistic regression and random forest models.

**Results:**

Among the 90 confirmed COVID-19 cases, 79 were mild and 11 were severe. The average age of the patients was 36.1 years old, including 49 males and 41 females. The average age of severe patients is significantly older than that of mild patients (53.2 years old *vs* 33.7 years old). The average time from illness onset to hospital admission was 4.1 days and the average actual hospital stay was 18.7 days, both of these time actors were longer for severe patients than for mild patients. Forty-eight of the 90 patients (53.3%) had family cluster infections, which was similar among mild and severe patients. Comorbidities of underlying diseases were more common in severe patients, including hypertension, diabetes and other diseases. The most common symptom was cough [45 (50%)], followed by fever [43 (47.8%)], headache [7 (7.8%)], vomiting [3 (3.3%)], diarrhea [3 (3.3%)], and dyspnea [1 (1.1%)]. The laboratory findings of patients also included leukopenia [13(14.4%)] and lymphopenia (17.8%). Severe patients had a low level of creatine kinase (median 40.9) and a high level of D-dimer. The median NLR of severe patients was 2.82, which was higher than that of mild patients. Logistic regression showed that age, phosphocreatine kinase, procalcitonin, the lymphocyte count of the patient on admission, cough, fatigue, and pharynx dryness were independent predictors of COVID-19 severity. The classification of random forest was predicted and the importance of each variable was displayed. The variable importance of random forest indicates that age, D-dimer, NLR (neutrophil to lymphocyte ratio) and other top-ranked variables are risk factors.

**Conclusion:**

The clinical symptoms of COVID-19 patients are non-specific and complicated. Age and the time from onset to admission are important factors that determine the severity of the patient’s condition. Patients with mild illness should be closely monitored to identify those who may become severe. Variables such as age and creatine phosphate kinase selected by logistic regression can be used as important indicators to assess the disease severity of COVID-19 patients. The importance of variables in the random forest further complements the variable feature information.

## Introduction

Coronavirus disease 2019 (COVID-19) is an ongoing pandemic caused by severe acute respiratory syndrome coronavirus 2 (SARS-CoV-2) (Zhu et al., 2020). The virus was isolated and the whole genome sequenced in a short time, and was further found to have high homology with SARS coronavirus and bat coronavirus sequences through phylogenetic analysis (Lu et al., 2020; Wu et al., 2020; Zhou et al., 2020). The emergence of SARS-CoV-2 caused a profound change all over the world.

SARS-CoV-2 is an enveloped-RNA coronavirus. Symptoms of COVID-19 are highly variable, from none to severe illness. The symptoms are primarily fever, fatigue and dry cough, while gastrointestinal symptoms are not common. Most severe cases develop dyspnea after one week, and rapidly progress to acute respiratory distress syndrome, septic shock, coagulation dysfunction, and metabolic acidosis that are difficult to correct (Guan et al., 2020; Huang et al., 2020a).

Therefore, learning from the clinical characteristics and influencing factors of patients with mild and severe COVID-19, monitoring the changes in the patient’s condition, and providing strategies for severe patients entering the intensive care unit (ICU) as soon as possible, which may contribute to reduce mortality and deliver proper care.

## Research Objects and Methods

### Research Objects

This study retrospectively included 90 COVID-19-infected patients admitted to the Ninth People’s Hospital of Dongguan City from January 21 to March 31, 2020.All patients were confirmed in accordance with the protocol of “Diagnosis and Treatment Plan for COVID-19 (Trial Version 7)” issued by the General Office of the National Health Commission and the Office of the State Administration of Traditional Chinese Medicine on February 19, 2020, and were detected by quantitative reverse transcription PCR (RT-QPCR), and both chest radiographs and nasopharyngeal swabs and were performed for all patients upon admission. This study was approved by the Ethics Committee of the Ninth People’s Hospital of Dongguan, China.

### Data Set Processing

The severity (mild and serious) of COVID-19 patients is defined according to the diagnosis and description of the disease course in the electronic medical record. The laboratory values of heart rate, the white blood cell count of the patient on admission (white blood cell count 1), the lymphocyte count of the patient on admission (lymphocyte count 1), the platelet count of the patient on admission (platelet count 1), and white blood cell count after admission (white blood cell count 2), lymphocyte count after admission (lymphocyte count 2), platelet count after admission (platelet count 2), NLR(Neutrophil to lymphocyte ratio), prothrombin time, phosphocreatine kinase, phosphocreatine kinase isoenzyme, procalcitonin, D-dimer were all measured. Some indexes lack data, so the random forest method is used for interpolation. The outcome of the patients were discharged from hospital.

The proportion of mild and severe patients is unbalanced (79 mild patients and 11 severe patients). The imbalance of categories will affect the modeling effect, the prediction effect of the classification model will be reduced, and the prediction result tends to favor the majority category. Therefore, the synthetic minority sample oversampling method (SMOTE) was used to balance the data. The SMOTE method is a data preprocessing technique applied to imbalance problems proposed by (Chawla et al., 2002). It uses the K-nearest neighbors and linear interpolation to add artificially synthesized minority class samples to the data, combined with the under-sampling method in the majority class, to balance the class distribution and reduce the possibility of overfitting. The SMOTE method operates in the feature space, so that the minority decision-making regions become more general. The SMOTE method can make the classifier build a larger decision-making area, including nearby minority class points, so it can improve the classification performance (Chawla et al., 2002). At present, SMOTE algorithm has been widely used in many fields, and SMOTE can effectively speed up classification, such as random forest, decision tree, Bayesian network, etc. Some studies have shown that the accuracy of these classification models using SMOTE training models is better than that of models not using SMOTE training (Alahmari, 2020).

The parameter percent.over in the minority sample was set to 1000, and percent.under for the majority sample was set to 100. This parameter setting means that the minority class will eventually generate 1+ percent.over/100 times the original, and the majority class will eventually be generated as (percent.under/100)*(percent.over/100) times the minority class. Finally got 110 mild cases and 121 severe cases in the model. Since the algorithm needs to extract samples from most types of samples, the data of mild and severe cases after balancing has increased.

### Research Methods

In this paper, descriptive statistical methods are used to study the clinical characteristics of mild and severe patients. A T-test was used for the data subject to normal distribution, a Wilcox rank sum test was used for the continuous skewed data, and a chi-square test was used to compare the differences between the two groups of data for the classified data. After the data is balanced, based on the Lasso-Logistic regression model to screen the risk factors and symptoms related to severe illness, a P value of less than 0.05 is considered statistically significant. This article use the Shapiro-Wilk (S-W) method to judge the normality of continuous variables.

Lasso regression is a kind of machine learning regression, which is used to select the important factors influencing the results. Leucopenia was defined as a white blood cell count below 4×10^9^/L; lymphocytopenia was defined as a lymphocyte count below 1×10^9^/L. The software used in this article is R 3.6.2, where the balance data is the SMOTE function in the DMwR package in the R language. The Lasso regression model uses the gelnet function in the R language. This paper also uses random forest for classification modeling. The model is a classification tree-based algorithm proposed by Breiman and Cutler, which improves the prediction accuracy of the model by summarizing a large number of classification trees. Compared with general regression, it is not sensitive to multivariable collinearity, and it is more robust to missing data and unbalanced data. At the same time, it can give the importance of variables, evaluate the role of each variable in classification, and make good predictions (Yang et al., 2020b). The model is implemented using the Random Forest function in R language.

## Results

### Basic Information, Epidemiological History and Clinical Manifestations

**Figure 1:**
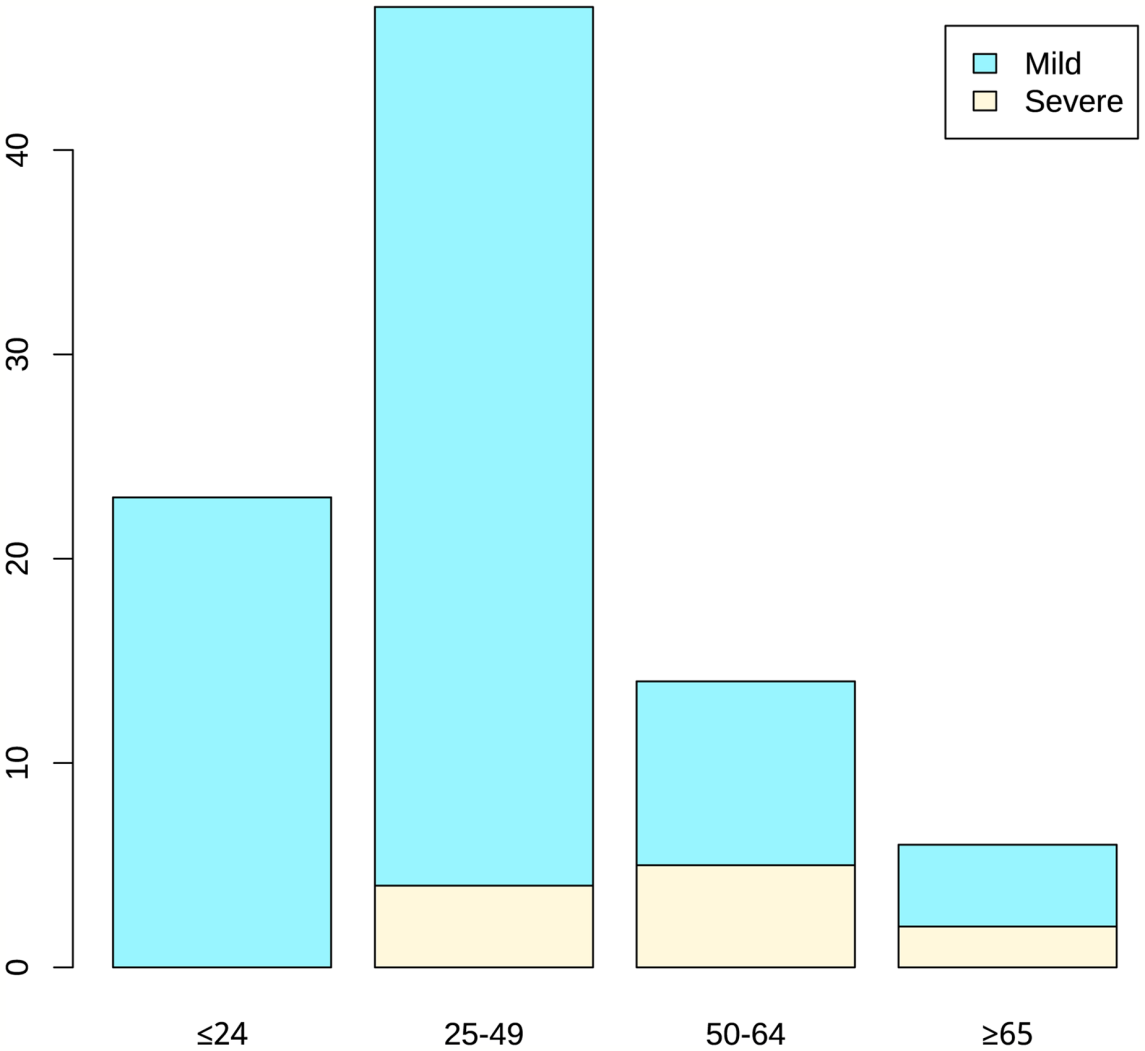
Age distribution of patients.

Among the 90 confirmed COVID-19 patients (49 males and 41 females), 79 patients were mild and 11 patients were severe. The average age of all patients was 36.1 years, the median was 36.5 years, and 50% of patients were between 21.3-47 years old. The age quartile of mild patients ranged from 19 to 45 years, while the severe patients ranged from 46 to 59.5 years. The mean age of severe patients was 53.1 years, which is significantly higher than the mean age of mild patients (33.7 years). With the increase of age, the proportion of serious patients with increased (P < 0.05). The average time from onset to admission was 4.1 days, with a median of 2.5 days. The average actual hospital stay was 18.7 days, with a median of 18 days. And the above two for severe patients were both longer compared to mild patients. Forty-eight of 90 patients (53.3%) had family cluster infections in severe patients, which was similar among in mild patients. Nearly half of the patients suffered from basic diseases [40 (44.4%)], mainly including hypertension [11 (12.2%)], chronic liver disease [7 (7.8%)] and other basic diseases [25 (27.8%)]. Basic diseases were more common in severe patients than mild ones, while hypertension, diabetes and other diseases were also more obvious. Eleven of the 90 patients were seriously ill, 8 of whom had underlying diseases, accounting for 72.7%. There were 79 patients with mild disease, among which 32 patients had underlying disease, accounting for 40.5%. On admission, most patients had fever [43 (47.8%)] or cough [45 (50%)]. Among them, 36(45.6%) of the mild patients had fever and 7(63.6%) of the mild patients had fever. There were 37 mild patients with cough (46.8%) and 8 severe patients with cough (72.7%). A total of 29 patients (32.2%) presented with both cough and fever. Other symptoms included dyspnea [1 (1.1%)], diarrhea [3 (3.3%)], emesis [3 (3.3%)], headache [7 (7.8%)]. (**Table 1**; **Figure 1**). This suggested that elderly patients with delayed diagnosis and admission for treatment were more likely to develop severe illnesses after being infected with COVID-19. (The continuous variables in the table have been tested for normality. Among them, age and actual hospital stay follow a normal distribution. Other variables use the median (quartile).

**Table 1:** Demographic and clinical characteristics of COVID-19 infected patients.

Basic characteristics		All patients (n=90)	Mild (n=79)	Severe (n=11)	*P* value
Gender [n]					
female		41	36	5	1
male		49	43	6	
age (mean (SD))(years)		36.07 (18.36)	33.70 (17.69)	53.09 (13.99)	<0.001
Time from onset to admission (d)		2.50 [1.00, 4.00]	2.00 [1.00, 4.00]	4.00 [2.50, 9.00]	0.016
Actual number of days hospitalized (mean (SD))(d)		18.67 (7.49)	18.53 (7.30)	19.73 (9.08)	0.682
Family gathering infection [n(%)]		48 (53.3)	41 (51.9)	7 (63.6)	0.683
Comorbidities [n(%)]					
Have disease		40 (44.4)	32 (40.5)	8 (72.7)	0.091
Hypertension		11 (12.2)	6 (7.6)	5 (45.5)	——
Diabetes		4 (4.4)	1 (1.3)	3 (27.3)	——
Cardiovascular diseases		3 (3.3)	2 (2.5)	1 (9.1)	——
Chronic liver disease		7 (7.8)	6 (7.6)	1 (9.1)	——
Respiratory disease		5 (5.6)	4 (5.1)	1 (9.1)	——
Nervous system disease		1 (1.1)	1 (1.3)	0	——
Metabolic diseases		4 (4.4)	4 (5.1)	0	——
Blood system diseases		0	0	0	——
Chronic kidney disease		2 (2.2)	2 (2.5)	0	——
Tumor		3 (3.3)	2 (2.5)	1 (9.1)	——
other		25 (27.8)	19 (24.1)	6 (54.5)	——
Signs and symptoms [n(%)]					
Fever		43 (47.8)	36 (45.6)	7 (63.6)	0.423
Cough		45 (50)	37 (46.8)	8 (72.7)	0.198
Expectoration		23 (25.6)	18 (22.8)	5 (45.5)	0.213
Fatigue		10 (11.1)	8 (10.1)	2 (18.2)	0.776
Dyspnea		1 (1.1)	0	1 (9.1)	0.246
Diarrhea		3 (3.3)	2 (2.5)	1 (9.1)	0.811
Poor appetite		12 (13.3)	10 (12.7)	2 (18.2)	0.975
Emesis		3 (3.3)	2 (2.5)	1 (9.1)	0.811
headache		7 (7.8)	6 (7.6)	1 (9.1)	1
Muscle ache		10 (11.1)	9 (11.4)	1 (9.1)	1
Pharynx dryness		13 (14.4)	9 (11.4)	4 (36.4)	0.080

**Table 1-2** showed the demographic and clinical characteristics of the patients after the SMOTE balance. In terms of the difference indicators of the patients with mild and severe diseases, the significant variables, except age and the time from onset to admission, were newly added with basic diseases, fever, cough, expectoration, fatigue and pharynx dryness.

**Table 1-2:** The demographic and clinical characteristics of patients infected with COVID-19 after SMOTE balance data.

Basic characteristics		All patients (n=231)	Mild (n=110)	Severe (n=121)	*P* value
Gender [n]					
	Female	130	57	73	0.242
	Male	101	53	48	
age(years)		45.00[35.00,56.14]	35.00[20.00,41.00]	53.43[47.52,59.23]	<0.001
Time from onset to admission (d)		3.00 [2.00, 6.32]	2.00 [1.00, 4.00]	4.00 [2.51, 8.83]	<0.001
Actual number of days hospitalized (d)		19.00[14.00,23.00]	18.00[13.00,23.00]	20.36[14.56,23.28]	0.697
Family gathering infection [n(%)]		125 (54.1)	57 (51.8)	68 (56.2)	0.593
Comorbidities [n(%)]					
	Have disease	134 (58.0)	46 (41.8)	88 (72.7)	<0.001
	hypertension	52 (22.5)	3 (2.7)	49 (40.5)	——
	diabetes	16 (6.9)	3 (2.7)	13 (10.7)	——
	Cardiovascular diseases	8 (3.5)	2 (1.8)	6 (5.0)	——
	Chronic liver disease	24 (10.4)	7 (6.4)	17 (14.0)	——
	Respiratory disease	12 (5.2)	6 (5.5)	6 (5.0)	——
	Nervous system disease	0	0	0	——
	Metabolic diseases	4 (1.7)	4 (3.6)	0 (0.0)	——
	Blood system diseases	0	0	0	——
	Chronic kidney disease	3 (1.3)	3 (2.7)	0 (0.0)	——
	Tumor	9 (3.9)	3 (2.7)	6 (5.0)	——
	other	100 (43.3)	29 (26.4)	71 (58.7)	——
Signs and symptoms [n(%)]					
	Fever	109 (47.2)	40 (36.4)	69 (57.0)	0.003
	Cough	149 (64.5)	44 (40.0)	105 (86.8)	<0.001
	Expectoration	93 (40.3)	18 (16.4)	75 (62.0)	<0.001
	Fatigue	33 (14.3)	8 (7.3)	25 (20.7)	0.007
	Dyspnea	6 (2.6)	0 (0.0)	6 (5.0)	0.051
	Diarrhea	11 (4.8)	3 (2.7)	8 (6.6)	0.282
	Poor appetite	39 (16.9)	16 (14.5)	23 (19.0)	0.466
	Emesis	8 (3.5)	2 (1.8)	6 (5.0)	0.345
	headache	22 (9.5)	6 (5.5)	16 (13.2)	0.074
	Muscle ache	16 (6.9)	11 (10.0)	5 (4.1)	0.135
	pharynx dryness	64 (27.7)	10 (9.1)	54 (44.6)	<0.001

### Laboratory Examination Indexes

The statistical results of laboratory examination indexes of 90 patients were shown in **Table 2**. There was no significant difference in the heart rate among with mild to severe cases upon admission. On admission, the blood count of the patients showed leukopenia in 13 of them (14.4%), lymphocytosis in 16 of them (17.8%), and thrombocytopenia in 0. After a period of treatment, the blood count of the patients measured during the hospitalization showed that there were 6 patients with leukopenia (6.67%), 4 patients with lymphopenia (4.44%), and 2 patients with thrombocytopenia (2.22%). These indicators were not statistically significant between mild and severe patients (*P*>0.05). The median time of development of prothrombin was 12.5 in both mild and severe cases, and the level of procalcitonin was also similar. Levels of phosphocreatine kinase were low in critically ill patients (The median of creatine phosphokinase was 40.9). The D-dimer level was higher in severe patients than in mild patients, but there was a difference in severe patients *(P* < 0.05). The NLR level of severe patients is higher than that of mild patients. The median NLR of severe patients was 2.82, which was higher than the median of 2.44 for mild patients. There is no significant difference between the two groups in the original data. After class-balanced data, NLR is significant(P=0.048). The treatment plan was based on antiviral and anti-infective treatment, and severe patients used oxygen therapy more than mild patients. **Table 2-2** showed the results of laboratory examinations and treatment plans for patients after the class balance. In terms of different indicators for mild and severe patients, in addition to D-dimer, the newly added (just admitted) lymphocytes count, procalcitonin, NLR, (after admission) platelet count, creatine phosphate kinase.

**Table 2:** Laboratory test results and treatment regimens for COVID-19 patients during hospitalization.

Laboratory index		All patients (n = 90)	Mild (n = 79)	Severe (n = 11)	*P* value
Heart rate (mean (SD))(Times/min)		90.68 (12.98)	90.73 (12.83)	90.27 (14.66)	0.923
white blood cell count 1 (×10^9^/L)		5.26 [4.60, 6.38]	5.25 [4.61, 6.39]	5.75 [4.64, 6.21]	0.936
<4		13(14.4%)	11(13.9%)	2(18.2%)	——
4-10		74(82.2%)	66(83.5%)	8(72.7%)	——
>10		3(3.33%)	2(2.5%)	1(9.1%)	——
Lymphocyte count 1(×10^9^/L)		1.33 [1.05, 1.98]	1.39 [1.06, 2.00]	1.11 [0.89, 1.52]	0.128
	<1.0	16(17.8%)	13(16.5%)	3(27.3%)	——
	≥1.0	74(82.2%)	66(83.5%)	8(72.7%)	——
Platelet count 1(×10^9^/L)		179.00 [164.00, 237.00]	179.00 [162.00, 237.00]	201.00 [171.00, 224.00]	0.54
	<100	0	0	0	——
	≥100	90	79	11	——
White blood cell count 2(×10^9^/L)		5.49 [4.92, 6.78]	5.52 [4.96, 6.78]	5.04 [4.74, 6.75]	0.378
	<4	6 (6.67%)	4(5.1%)	2(18.2%)	——
	4-10	83(92.2%)	74(93.7%)	9(81.8%)	——
	>10	1(1.11%)	1(1.3%)	0	——
Lymphocyte count 2 (×10^9^/L)		1.57 [1.37, 1.97]	1.58 [1.38, 1.98]	1.46 [1.23, 1.73]	0.232
	<1.0	4(4.44%)	3(3.8%)	1(9.1%)	——
	≥1.0	86(95.6%)	76(96.2%)	10(90.9%)	——
Platelet count 2 (×10^9^/L)		221.34 [198.75, 271.25]	221.54 [196.00, 273.00]	217.20 [211.30, 233.64]	0.966
	<100	2(2.22%)	2(2.5%)	0	——
	≥100	88(97.8%)	77(97.4%)	11(100%)	——
Prothrombin time (s)		12.50 [12.30, 12.99]	12.50 [12.30, 13.11]	12.50 [12.31, 12.86]	0.777
Creatine phosphate kinase		43.86 [37.59, 59.90]	44.74 [37.98, 61.00]	40.91 [36.51, 51.56]	0.542
	<50	54(60%)	46(58.2%)	8(72.7%)	——
	≥50 to <350	36(40%)	33(41.8%)	3(27.3%)	——
Creatine phosphate kinase isoenzyme		8.58 [7.39, 11.63]	8.90 [7.35, 12.05]	8.35 [7.45, 8.54]	0.254
Procalcitonin (ng/ml)		0.11 [0.08, 0.15]	0.11 [0.08, 0.14]	0.12 [0.10, 0.18]	0.158
	<0.1	34(37.8%)	32(40.5%)	2(18.2%)	——
	≥0.1 to <0.25	52(57.8%)	43(54.4%)	9(81.8%)	——
	≥0.25 to <0.5	4(4.44%)	4(5.1%)	0	——
D - dimer (μg/L)		0.47 [0.28, 0.67]	0.44 [0.24, 0.65]	0.75 [0.57, 1.04]	0.002
NLR		2.47 [1.84, 3.38]	2.44 [1.82, 3.38]	2.82 [2.34, 3.58]	0.213
Treatment options					
	Antiviral infection	86(95.6%)	75(94.9%)	11(100%)	——
	Anti-infective treatment	51(56.7%)	40(50.6%)	11(100%)	——
	Chinese medicine treatment	31(34.4%)	28(35.4%)	3(27.3%)	——
	oxygen therapy	38(42.2%)	28(35.4%)	10(90.9%)	——

The data were median.

**Table 2-2:** Laboratory test results and treatment plan of COVID-19 patients during hospitalization after SMOTE algorithm balancing data.

Laboratory index		All patients (n = 231)	Mild(n = 110)	Severe (n = 121)	*P* value
Heart rate (Times/min)		88.74 [82.00, 98.00]	90.00[82.00,103.75]	86.91[81.98,94.42]	0.075
white blood cell count 1 (×10^9^/L)		5.11 [4.27, 6.01]	5.03 [4.23, 6.01]	5.21 [4.44, 5.99]	0.799
<4		41(17.7%)	21(19.1%)	20(16.5%)	
4-10		185(80.1%)	87(79.1%)	98(81.0%)	
>10		5(2.2%)	2(1.8%)	3(2.5%)	
Lymphocyte count 1 (×10^9^/L)		1.28 [1.05, 1.74]	1.45 [1.05, 2.03]	1.23 [1.05, 1.60]	0.038
	<1.0	48(20.8%)	21(19.1%)	27(22.3%)	
	≥1.0	183(79.2%)	89(80.9%)	94(77.7%)	
Platelet count 1 (×10^9^/L)		179.00[168.80,216.93]	174.50[170.00,237.00]	184.86 [168.64, 208.51]	0.689
	<100	0	0	0	
	≥100	231(100%)	110(100%)	121(100%)	
white blood cell count 2 (×10^9^/L)		5.35 [4.78, 6.76]	5.39 [4.82, 6.76]	5.26 [4.74, 6.74]	0.315
	<4	15(6.5%)	2(1.8%)	13(10.7%)	
	4-10	215(93.1%)	107(97.3%)	108(89.3%)	
	>10	1(0.4%)	1(0.9%)	0	
Lymphocyte count 2 (×10^9^/L)		1.53 [1.36, 1.89]	1.54 [1.37, 1.94]	1.50 [1.35, 1.89]	0.101
	<1.0	3(1.3%)	1(0.9%)	2(1.7%)	
	≥1.0	228(98.7%)	109(99.1%)	119(98.3%)	
Platelet count 2 (×10^9^/L)		229.20 [212.57, 283.81]	224.00 [198.75, 273.50]	233.61 [215.42, 288.51]	0.016
	<100	3(1.3%)	3(2.7%)	0	
	≥100	228(98.7%)	107(97.3%)	121(100%)	
Prothrombin time (s)		12.51 [12.31, 12.95]	12.48 [12.31, 12.89]	12.57 [12.31, 12.95]	0.703
Creatine phosphate kinase		44.71 [38.12, 68.08]	43.00 [37.51, 62.97]	45.19 [38.88, 76.26]	0.035
	<50	131(56.7%)	65(59.1%)	66(54.5%)	
	≥50 to <350	100(43.3%)	45(40.9%)	55(45.5%)	
Creatine phosphate kinase isoenzyme		8.47 [7.81, 10.07]	8.58 [7.43, 10.97]	8.44 [8.03, 9.38]	0.735
Procalcitonin (ng/ml)		0.12 [0.09, 0.17]	0.11 [0.08, 0.14]	0.14 [0.10, 0.18]	<0.001
	<0.1	63(27.3%)	39(35.5%)	24(19.8%)	
	≥0.1 to<0.25	165(71.4%)	68(61.8%)	97(80.2%)	
	≥0.25 to<0.5	3(1.3%)	3(2.7%)	0	
D - dimer (μg/L)		0.62 [0.46, 0.95]	0.47 [0.29, 0.62]	0.90 [0.61, 1.10]	<0.001
NLR		2.55 [2.07, 3.39]	2.46 [1.84, 3.46]	2.62 [2.12, 3.36]	0.048
Treatment programs					
	Antiviral infection	225 (97.4)	104 (94.5)	121 (100.0)	——
	Anti-infective treatment	172 (74.5)	51 (46.4)	121 (100.0)	——
	Chinese medicine treatment	66 (28.6)	37 (33.6)	29 (24.0)	——
	oxygen therapy	135 (58.4)	33 (30.0)	102 (84.3)	——

The data were median.

### Lasso-Logistics Regression Model

The dependent variable was severity. Severe was 1 and mild was 0. There were a total of 30 independent variables, including population basic information (age, gender), hospital medical detection (heart rate), time from onset to admission, actual number of days hospitalized, blood routine examination on admission (white blood cell count 1, lymphocyte count 1, platelet count 1), hospitalization, blood routine examination (white blood cell count 2, lymphocyte count 2, platelet count 2) and blood coagulation (prothrombin time, D-dimer), NLR(Neutrophil to lymphocyte ratio), myocardial enzymes including creatine phosphate kinase (CK) and creatine phosphate kinase isoenzyme (CK-MB), myocardial markers (calcitonin former), underlying disease, family gathering infection, and symptoms (fever, cough, expectoration, fatigue, dyspnea, diarrhea, poor appetite, emesis, headache, muscle aches, pharynx dryness). Some variable names are abbreviated as follows: Time from onset to admission (onset time), actual number of days hospitalized (Actual days), Family gathering infection (FG infection), Have underlying disease (HU disease), White blood cell count 1 (WBC1), Lymphocyte count 1 (LYMBH1), Platelet count 1 (PLT1), White blood cell count 2 (WBC2), Lymphocyte count 2 (LYMBH2), Platelet count 2 (PLT2), creatine phosphate kinase (CK), creatine phosphate kinase isoenzyme (CK MB).

The significance of the variables under the univariate logistic regression was calculated first. The results were shown in **Table 3**. The significant variables were age, underlying disease, fever, cough, expectoration, fatigue, pharynx dryness, time from onset to admission, creatine phosphate kinase, procalcitonin, D-dimer, lymphocyte count 2 and lymphocyte count 1.

**Table 3:** Significance of single-factor logistic regression variables.

Variable name	Gender (Female)	Age	Underlying disease	Heart rate	Fever	Cough
P value	0.193	5.54e-14***	3.11e-06***	0.0635	0.00182**	5.27e-12***
Variable name	Expectoration	Fatigue	Dyspnea	Diarrhea	Poor appetite	Emesis
P value	2.83e-11***	0.0053**	0.987	0.180	0.367	0.211
Variable name	headache	Muscle ache	FG_infection	Pharynx dry	onset_time	WBC2
P value	0.0513	0.0888	0.505	3.59e-08***	0.00657**	0.230
Variable name	LYMBH2	PLT2	Prothrombin time	CK	CK-MB	Procalcitonin
P value	0.0355*	0.0737	0.0784	0.00156**	0.824	2.94e-06***
Variable name	D-dimer	Actual_days	WBC1	LYMBH1	PLT1	NLR
P value	5.96e-07***	0.832	0.488	0.01005*	0.1224	0.804

*indicates that the P value of the variable is less than 0.05. **indicates that the P value is less than 0.01. ***indicates that the P value is less than 0.001.

**Figure 2:**
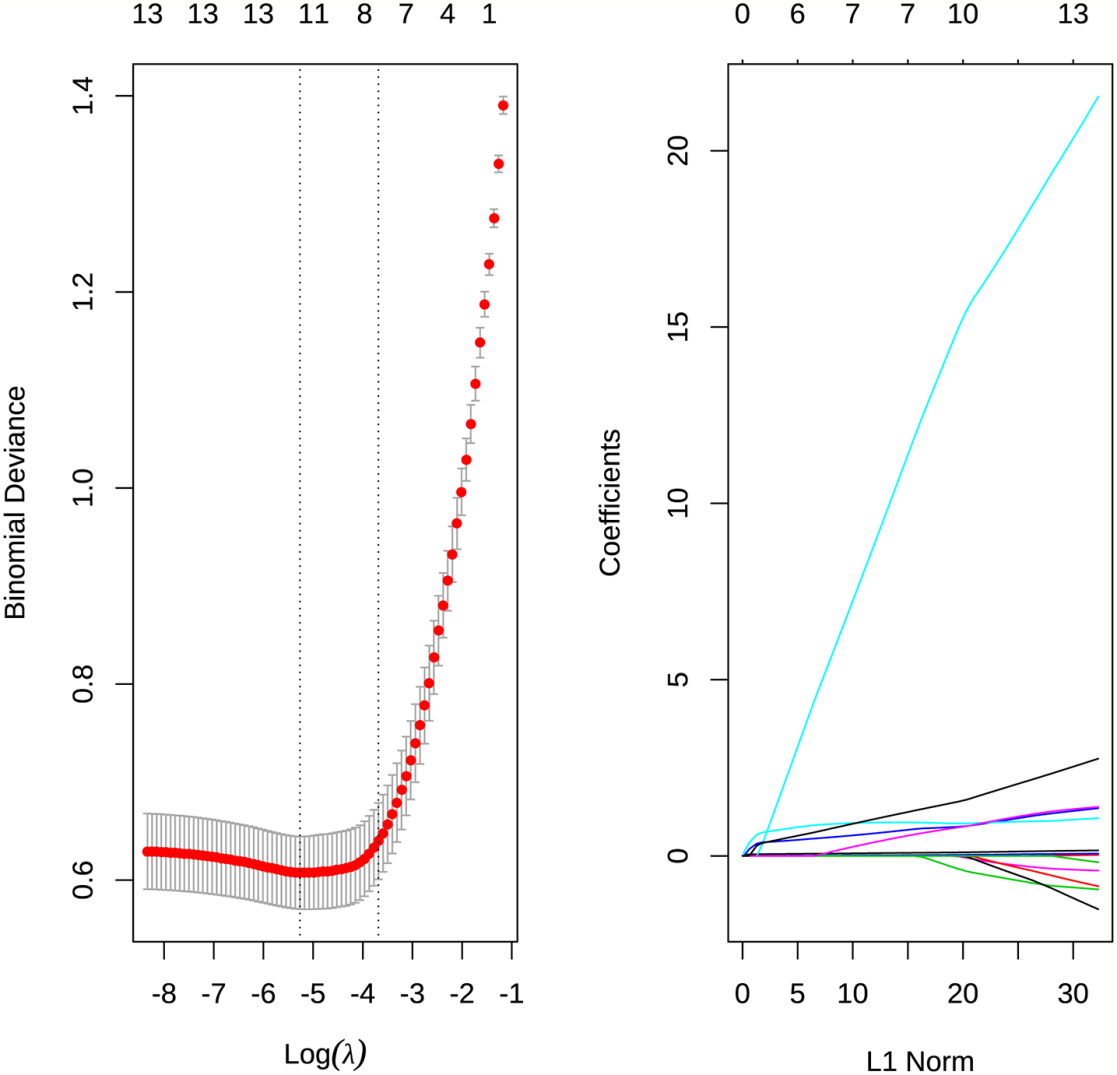
**(A)** Select the best λ value for the AUC value when compressing different variables. **(B)** The degree of compression of each variable under different penalty parameters λ.

The above variables were substituted into the Lasso regression. The complexity of Lasso was controlled by lambda, and the model obtained by different lambda was different. **Figure 2A** showed that the best lambda value can be selected according to the AUC value when compressing different variables. In **Figure 2B**, each colored line represented one variable, and the horizontal axis represented the L1 norm, which was the sum of the absolute values of the regression coefficients. The vertical axis represented the coefficient. If a vertical dashed line was drawn in the figure, the vertical line represented a penalty value, and the variable that intersects the color line was the selected variable.

According to the results of Lasso, the variables including age, CK, procalcitonin, D-dimer, LYMBH1, LYMBH2, underlying disease, fever, cough, expectoration, fatigue, pharynx dryness were selected as important variables. These variables were classified into the logistics model, and variables with insignificant P values were eliminated according to significance. The results were obtained (**Table 4**). Age, CK, procalcitonin, LYMBH1, cough, fatigue, and pharynx dryness were important variables.

**Table 4:** Logistics model results.

	Estimate	Std. Error	P value
(Intercept)	-13.42683	2.34558	1.04e-08***
Age	0.14735	0.02545	7.08e-09***
CK	0.06749	0.01521	9.17e-06***
Procalcitonin	24.92632	6.64398	0.000176***
LYMBH1	-1.62463	0.76513	0.033727*
Cough	1.84101	0.54942	0.000806***
Fatigue	1.64915	0.73679	0.025201*
Pharynx dryness	2.84222	0.76075	0.000187***

*indicates that the P value of the variable is less than 0.05.

***indicates that the P value is less than 0.001.

It can be seen from the results that, except for the constant term and LYMBH1, the coefficients of the other variables were all positive. Among them, age, CK, LYMBH1 and procalcitonin were continuous variables. Cough, fatigue and pharynx dryness were categorical variables. The results were tested for collinearity. VIF values were all less than 5. And there was no collinearity among the variables.

**Figure 3:**
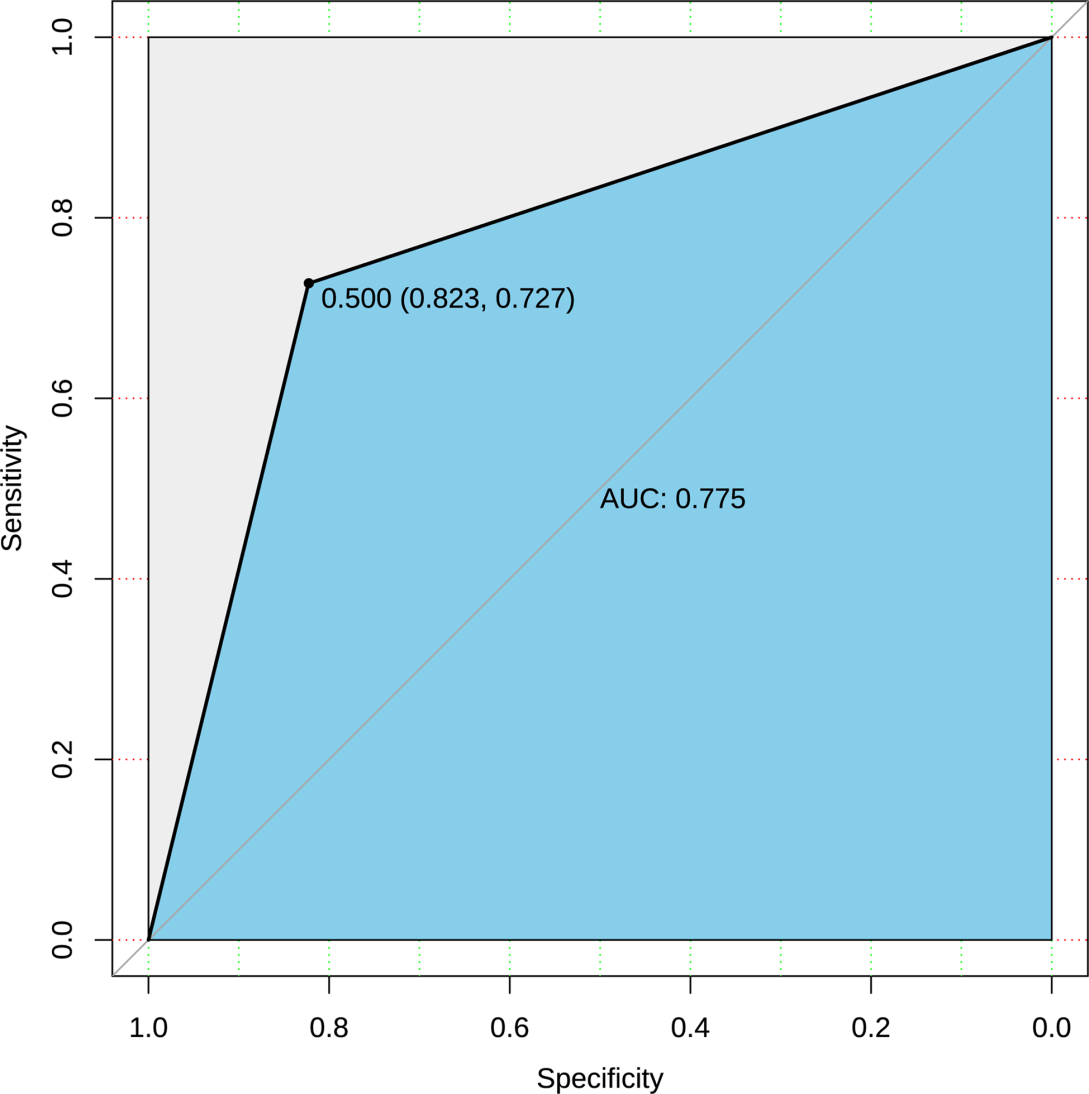
ROC curve of prediction based on raw data.

The logistic regression model was obtained according to the balanced data, and prediction accuracy was 81.1% when the original data was substituted into the model. The ordinate of ROC curve (**Figure 3**) was the true positive rate (sensitivity), and the abscissa was the false positive rate (1-specificity). The ideal point on the ROC curve was (0,1), which means all positive classes were correctly classified and all negative classes were not mistakenly classified as positive classes. Therefore, the closer the ROC point was to the upper left corner, the better the performance. In this article, the abscissa of the ROC graph is specific, and the ideal point is (1,1). The closer the AUC value of the area under the ROC curve to 1, the better the diagnostic effect. The coordinates of the best critical point on the curve in **Figure 3** were (0.823, 0.727), and the threshold was 0.5. It has high sensitivity and specificity at this point. The AUC value in the figure was 0.775. It showed that the accuracy of the model is good 

### Random Forest

Random forest was used to extract a certain number of self-service samples from the original data using the bootstrap method with replacement and a decision tree was built for each sample. At each node, among all competing independent variables, several random competitive split variable were selected. Each tree in the random forest was left unpruned and allowed to grow fully. The final prediction result was a simple average of the results of all decision trees (Moghadas et al., 2020). In this paper, the random forest method was used to classify variables, give the importance of variables, and make prediction.

**Figure 4:**
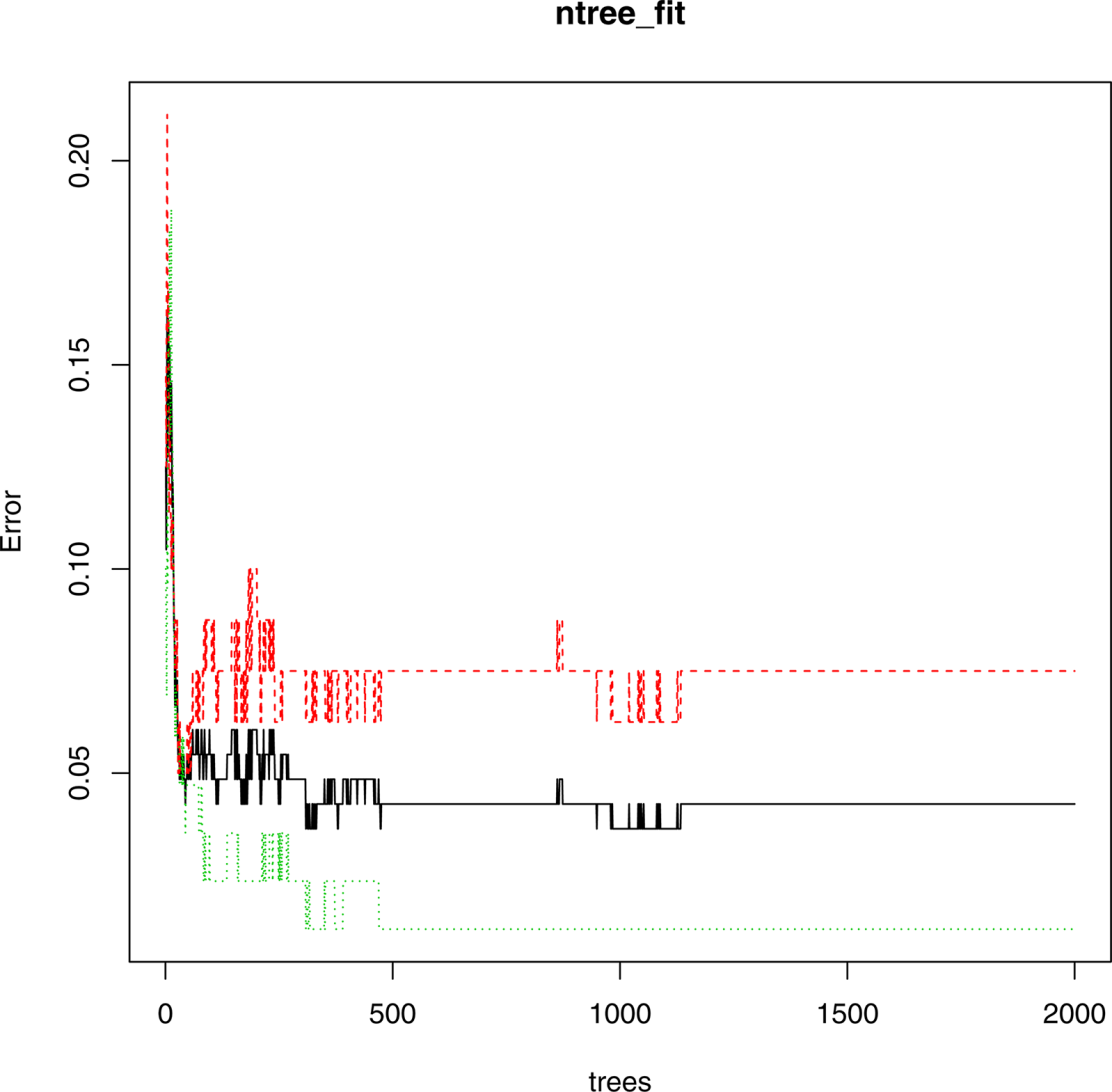
The false positive rate of each tree in the random forest.

The data was extracted by 7:3 for the training set and the test set. For the node binary tree parameter mtry, the loop statement was used to calculate the mean value of the misjudgment rate of all models, and the optimal mtry was 6. It can be seen from **Figure 4** that when the number of decision trees contained in the random forest ntree was about 1300, the three lines tended to be stable, that is, the error within the model was basically stable, so ntree=1300. At this time, the OOB error is 5.45%. It can be seen from **Table 5** that 74 patients with mild illness and 82 patients with severe illness were judged correctly, but still with a certain probability of misjudgment, and 6 patients with mild illness were judged as severe. In general, the correct rate of the model, that was, the proportion of samples that were correctly identified by all samples was (74 + 82)/165 = 94.54%; the accuracy mild rate indicates how many of the guessed positive samples are correct, and the mild disease was regarded as the positive sample. The precision rate was 74/(74 + 3)=96.1%; the recall rate indicates how many positive samples were recalled among all the positive samples, so the recall rate was 74/(74 + 6)=92.5%.

**Table 5:** Confusion matrix.

	Mild	Severe	class.error
Mild	74	6	0.07500000
Severe	3	82	0.03529412

**Table 6** showed the importance of variables, in which Mean Decrease Accuracy represents an average Decrease in Accuracy, and Mean Decrease Gini represents an average Decrease in node unpurity. A larger value indicates a stronger importance of variables. It can be seen from the table that age, D-dimer, time from onset to admission and other large values were important variables.

**Table 6:** Importance of variables.

Sequence	Variable name	Mild	Severe	Mean Decrease Accuracy	Mean Decrease Gini
1	Gender	0.000842276	0.000304033	0.000571913	0.148250306
2	Age	0.114189754	0.147716116	0.130207653	18.01285314
3	HU_disease	0.002974213	0.003116931	0.003079772	0.367923962
4	Heart rate	0.012114962	0.019990241	0.016000088	2.846081568
5	Fever	0.003780202	0.000847247	0.002318694	0.389517379
6	Cough	0.017572645	0.015699049	0.016538291	3.237108144
7	Expectoration	0.03154458	0.015864688	0.023332413	3.984819339
8	Fatigue	0.001708698	0.001729405	0.001704126	0.338710957
9	Dyspnea	0.000337112	0.000361101	0.000348378	0.198804768
10	Diarrhea	0.00045062	0.000512224	0.000479591	0.133668522
11	Poor appetite	0.0017915	0.001509113	0.001652048	0.273898309
12	Emesis	0.000103448	-2.91E-05	4.04E-05	0.02585151
13	headache	0.000741436	0.000554534	0.000661425	0.280146418
14	Muscle ache	0.000738661	0.00234859	0.001551771	0.328014275
15	FG_infection	0.00134232	0.000961904	0.001115436	0.196406068
16	pharynx dryness	0.013076183	0.012061961	0.012526584	2.197295907
17	Onset_time	0.027652778	0.05316072	0.040377103	6.231243065
18	WBC2	0.006230574	0.010898642	0.008580465	2.048265
19	LYMBH2	0.009000937	0.016263749	0.012845822	2.371037233
20	PLT2	0.013663865	0.017568322	0.015517789	2.663186165
21	Prothrombin_time	0.009546072	0.019385667	0.014444887	2.49562609
22	cK	0.012179427	0.02243054	0.017384287	2.454430363
23	cK-MB	0.008558614	0.019866388	0.014431234	2.067444666
24	Procalcitonin	0.016416281	0.033016345	0.024915001	3.630346879
25	D-dimer	0.063398879	0.089809009	0.076390526	12.2980474
26	Actual days	0.011318214	0.020510316	0.015964144	2.433158984
27	WBC1	0.013069936	0.019856579	0.016628493	2.610376896
28	LYMBH1	0.008186132	0.015641668	0.011974092	1.797733889
29	PLT1	0.012741624	0.021607278	0.017247375	2.606923867
30	NLR	0.012406788	0.025559522	0.019054742	3.198992108

**Figure 5:**
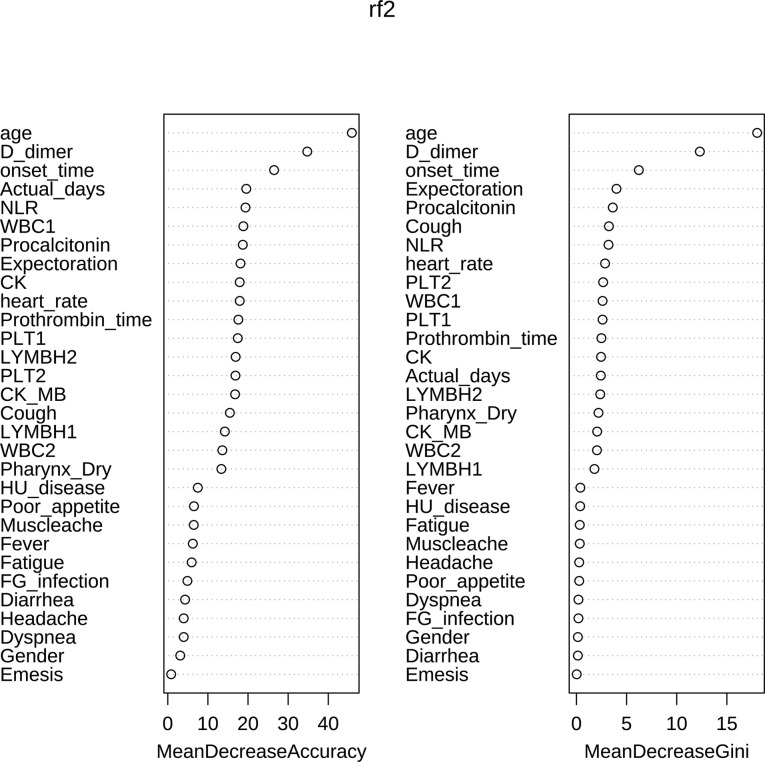
Ranking chart of importance of variables.

After sorting the Mean Decrease Accuracy and Mean Decrease Gini of the variables, **Figure 5** showed the visualization results of the variable importance ranking. According to the results of MeanAccuracy, we can see that the top 10 important variables were age, D dimer, onset time, Actual days, NLR, WBC1, procalcitonin, expectoration, CK, heart rate. According to the results of MeanDecreaseGini, we can see that the top 10 important variables were age, D dimer, onset time, expectoration, procalcitonin, cough, NLR, heart rate, PLT2, WBC1. Important variables in these two selection criteria include 8 variables: age, D dimer, onset time, procalcitonin, NLR, WBC1, expectoration, heart rate.

**Figure 6:**
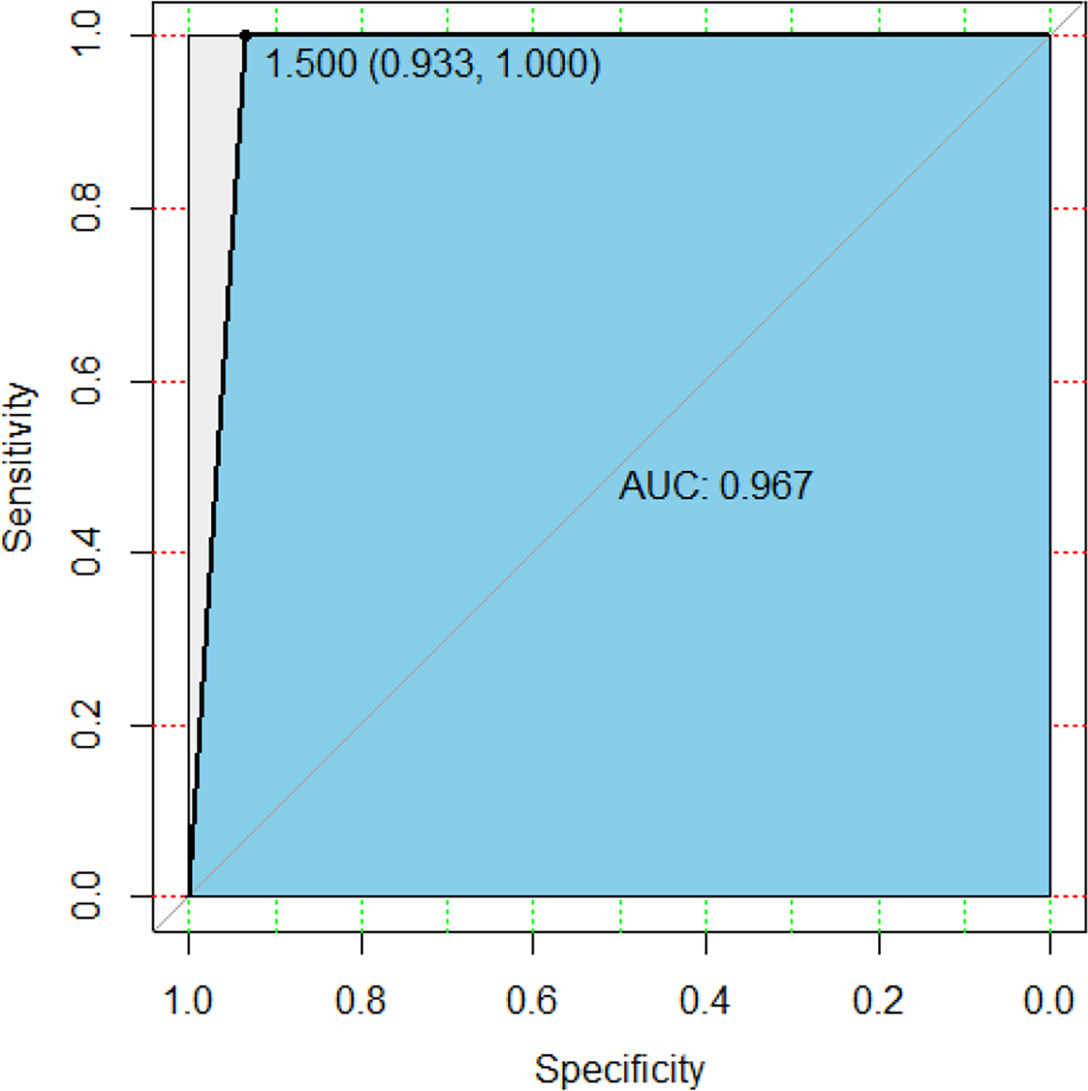
ROC curve and AUC area of random forest.

The prediction function was further used to verify the test set, and the model accuracy was 96.97%, the Sensitivity rate was 93.33%, and the recall rate was 93.3%. **Figure 6** was a random forest ROC curve and AUC evaluation graph. The optimal critical point threshold of the ROC curve was 1.5, and the coordinates are (0.933, 1), very close to the upper left. The AUC score under the ROC curve was 0.967 and the overall area of the curve was close to 1, indicating a strong recognition ability. All these indicate that the random forest method has a good effect.

## Discussion

Looking back on the past 20 years, the world has experienced two severe beta coronavirus pandemics, SARS-CoV in 2002-2003 and MERS-CoV in 2012. They had a fatality rate of 10% and 35% respectively (Drosten et al., 2003; Chen B. et al., 2020), which dealt a huge blow to public health. In early 2020, SARS-CoV-2 causing COVID-19 spread rapidly worldwide. At the same time, we need to be wary of the superposition of influenza and COVID-19 in the winter.

In this study, the epidemiological history, clinical characteristics and laboratory test indicators of 90 SARS-COV-2 nucleic acid positive patients with severe and mild illness were analyzed. The clinical manifestations are non-specific, mainly fever and cough. A small number of patients appeared breathing difficulties. Gastrointestinal symptoms are not common, which has also been confirmed by similar studies (Chen N. et al., 2020; Li et al., 2020). The incubation period of SARS-CoV-2 infection is longer than that of SARS and MERS. The viral load of asymptomatic infected patients is similar to that of symptomatic infected patients, which may lead to the underestimation the potential transmissibility of SARS-CoV-2 among people (Zou et al., 2020). More studies have shown that the invisible transmission of the COVID-19 is caused by the incubation period and asymptomatic infection (Moghadas et al., 2020; Wang et al., 2020). This is also one of the reasons why it is difficult to effectively control asymptomatic infection in the COVID-19 epidemic. Our data suggested that clinical monitoring should not just focus on fever symptoms. Otherwise, it will increase the missed diagnosis rate. It is necessary to strictly implement nucleic acid testing and be assisted by comprehensive judgment on clinical chest radiographs (Ai et al., 2020). Tracing and early isolation of asymptomatic infected persons are two of the key points for pandemic control (Grasselli et al., 2020). In addition, there is a serious family cluster infection in this study, which also suggests that we should be on guard against human-to-human transmission of the virus in families and hospitals, even inter-city and overseas transmission. It also reflects the importance of timely isolation of the source of infection and isolation at home in the early stage of the epidemic.

In this study, logistic regression presents seven important factors that influence the risk of severe COVID-19, which may be of great value in screening patients with mild or severe disease. Clinicians can quickly assess what kind of risk a patient is at. The seven variables are age, creatine kinase, procalcitonin, lymphocyte count 1, cough, fatigue and pharynx dryness.

In the COVID-19 epidemic, the high-risk groups affected are mainly the elderly and people with underlying diseases. Taking into account the decline in the rehabilitation ability of the elderly, the older patients are more likely to develop into severe patients (Hendren et al., 2020). The myocardial enzymes of patients with severe COVID-19 increased significantly, and the abnormal rate of myocardial enzymes of severe COVID-19 was higher than other groups (Babapoor-Farrokhran et al., 2020). Creatine phosphokinase is a kind of myocardial enzymes. Myocardial enzymes refer to a class of enzymes that catalyze the metabolism of myocardial cells and regulate the electrophysiological activities of myocardial cells. Once the cardiomyocytes are ruptured and necrotic, these enzymes are released into the blood and their values increase. Therefore, the changes in the myocardial enzyme indicators can measure the degree of damage to the cardiomyocytes. Especially in the early stage of myocardial infarction, when the myocardium has not yet undergone extensive necrosis, creatine kinase can be detected in human blood. Therefore, if this index is elevated, the degree of myocardial damage can be diagnosed in time (Babapoor-Farrokhran et al., 2020). Procalcitonin (PCT) is a biological marker that can be used to assess the possibility of bacterial infection, reflecting the severity of bacterial infection (Dymicka-Piekarska and Wasiluk, 2015). Different diseases have different concentrations of procalcitonin. The higher the concentration of procalcitonin, the higher the possibility of organ failure and the higher the risk of death. Severe disease in patients with COVID-19 is also closely related to lymphocytes. Studies have shown that lymphocytes in patients with COVID-19 show a significant decreasing trend, among which CD4+ and CD8+T lymphocytes are the most obvious. Therefore, CD4+ and CD8+T lymphocytes can be considered as early warning signals and prognostic indicators to judge the severity of COVID-19 patients (Huang et al., 2020b; Zhang et al., 2020). Three clinical symptoms, including cough, fatigue and pharyngeal dryness can be used as important clinical symptoms to judge the degree of disease development. Combined with the patient’s laboratory indicators, they have a certain reference.

The random forest classification method solves the problem of variable collinearity, and has a better prediction effect. At the same time, it also provides important predictors in the method, such as the age, D-dimer, time from onset to admission, procalcitonin, NLR, heart rate, white blood cell count. Among these variables, in addition to the age and procalcitonin variables explained above, D-dimer and NLR also have an important position. In this epidemic, some severe patients died because of an inflammatory storm, which is an overreaction of the human immune system. D-dimer is one of the markers for detecting thrombus, and the rise of D-dimer can also be seen when the human body undergoes inflammation (Shah et al., 2020). NLR is the ratio of neutrophils to lymphocytes. Neutrophils are the main component of the white blood cell population. After microbial pathogens invade the body, they will quickly reach the inflammation site and exert phagocytosis. In addition, the human immune response triggered by viral infections mainly relies on lymphocytes. In the previous logistic regression, it was also analyzed that the lymphocytes decreased, the higher the possibility of the patient being severely ill (Yang et al., 2020a; Kerboua, 2021). Therefore, elevated D-dimer and NLR indicate that the patient’s condition is getting worse. In this article, the D-dimer and NLR of severe patients are higher than those of mild patients. Random forests can still maintain accuracy for data with unbalanced classification and have a strong anti-overfitting ability. Therefore, the importance of variables given by random forests is further added to the variable information in addition to the logistics model. In the future, it can be considered as a direction in which to continue to look for key variables to distinguish the mild and severe diseases.

In this study, the clinical characteristics and modeling significant variables of patients with mild and severe diseases are of great value, and can help to identify whether or not the patients are at risk of critical illness in the early stage. However, there are some limitations in this study. Firstly, the clinical symptoms or signs and laboratory test results of patients extracted from electronic medical records lack some data, and some cases are incomplete. Secondly, because the research subjects are all hospitalized patients, the actual research lacks those with asymptomatic infections and mild patients who are not treated in hospital, causing the research results may be more inclined to serious outcomes. Thirdly, the sample size is limited, so the results may not be robust enough. The SMOTE algorithm performs class balancing and has certain errors. For example, it may repeatedly use outliers or wrong values in the data, or may incorrectly strengthen the local chance, thereby increasing the risk of overfitting. In addition, during the data collection period of this article, the Ninth People’s Hospital of Dongguan City had no moderate cases, so patients were divided into two types.

To sum up, the COVID-19 outbreak in 2020 is a huge public health crisis to the whole world. In the context of the pandemic, early detection, early isolation and early intervention have always been the basic strategies for epidemic prevention and control. The data shows that we need to closely monitor patients with mild COVID-19, reduce the transformation from mild to severe, and rationally allocate medical resources in a scientific and efficient way to improve the cure rate of COVID-19.

## Data Availability Statement

The datasets and R language code presented in this study can be found at GitHub (https://github.com/johnsnowgo/covid-90data).

## Ethics Statement

The studies involving human participants were reviewed and approved by The Ethics Committee of the Ninth People’s Hospital of Dongguan, China.

## Author Contributions

XC: Conceptualization, Methodology, Writing-review & editing, Project administration. LZ: Software, Formal analysis, Writing - original draft, Writing - review & editing. SY and HZ: Resources, Conceptualization, Methodology, Writing - review & editing. YJ: Conceptualization, Methodology, Writing-review & editing, Project administration. Y-QC: Conceptualization, Methodology, Writing-review & editing, Project administration. MX: Writing-original draft, Formal analysis. KL: Writing-original draft, Formal analysis. YL: Writing-original draft, Formal analysis. All authors contributed to the article and approved the submitted version.

## Funding

This study was supported by the National Natural Science Foundation of China (11601083), the program for Probability and Statistics: Theory and Application (IRTL1704) and Innovative Research Team in Science and Technology in Fujian Province University (IRTSTFJ). This study was also supported by National Natural Science Foundation of China (31970881) and Shenzhen Science and Technology Program under Grant (SGG20200225152008136) to Y-QC. The special topic of prevention and control technology research and promotion of emergency tackling on Covid-19 in Dongguan city (2020717150119128).

## Conflict of Interest

The authors declare that the research was conducted in the absence of any commercial or financial relationships that could be construed as a potential conflict of interest.

## Publisher’s Note

All claims expressed in this article are solely those of the authors and do not necessarily represent those of their affiliated organizations, or those of the publisher, the editors and the reviewers. Any product that may be evaluated in this article, or claim that may be made by its manufacturer, is not guaranteed or endorsed by the publisher.
